# Microwave Radiation Associated with the Titanate Nanotubes Catalyst to PET Glycolysis

**DOI:** 10.1021/acsomega.4c11425

**Published:** 2025-06-23

**Authors:** Ananda Ramires das Neves Stigger, Tiago Vidaletti, Marcus Seferin, Wesley Formentin Monteiro, Mario Lucio Moreira, Rosane Angélica Ligabue

**Affiliations:** † Programa de Pós-Graduação em Engenharia e Tecnologia de Materiais—PGETEMA, Escola Politécnica, Pontifícia Universidade Católica do Rio Grande do Sul—PUCRS, Av. Ipiranga, 6681, Partenon, Porto Alegre 90619-900, RS, Brazil; ‡ Departamento de Química Inorgânica, Instituto de Química, Universidade Federal do Rio Grande do Sul—UFRGS, Av. Bento Gonçalves, 9500, Agronomia, Porto Alegre 90650-001, RS, Brazil; § Programa de Pós-Graduação em Física—PPGFís, Departamento de Física, 37902 Universidade Federal de Pelotas—UFPel, Campus Capão do Leão, Capão do Leão 96001-970, RS, Brazil

## Abstract

The pursuit of poly­(ethylene terephthalate) (PET) recycling procedures, particularly those employing gentler and more efficient methods, is essential. Chemical recycling by glycolysis is one of the alternatives for this process, showing several advantages, including recovery of the monomer (bis­(2-hydroxyethyl) terephthalate (BHET)). Therefore, the aim of this work is to chemically recycle PET (virgin and postconsumer) via glycolysis, using titanate nanotubes (TNT) as a reaction catalyst. The novelty of this work lies in the utilization of the microwave heating process, with the reaction occurring in a closed system. The results obtained showed high efficiency in terms of reaction time, reducing the total PET conversion time to 1 h and achieving a BHET yield close to 80%, with weight ratios of ethylene glycol (EG)/PET = 4:1 and PET/catalyst = 300:1; *T* = 196 °C.

## Graphical abstract

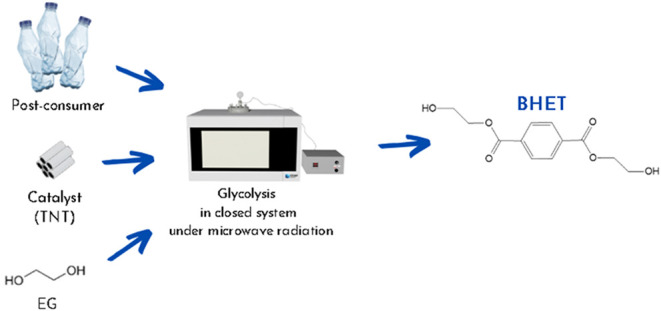

## Introduction

Polymeric materials are among the most used materials in everyday life. Poly­(ethylene terephthalate) (PET), for example, is a thermoplastic polymer belonging to the polyester family. It is widely used as beverage packaging because it has low manufacturing costs, high mechanical resistance, is light and flexible, and is also considered an inert material, making it safe for contact with food or drinks.^1–3^ However, improper disposal of PET bottles causes significant environmental problems. This issue is primarily due to the production of PET for single-use applications, making it a critical global concern.^4–6^

Therefore, recycling methods are essential to reduce waste in the environment. The process of recovering waste directly produced by factories is known as primary recycling. In this case, the material is reinserted into the industrial process without suffering contamination, since it does not come into contact with the final consumer.^7–9^ Secondary recycling, or mechanical recycling, is one of the types of recycling used for postconsumer PET waste. In this process, recycled PET is used to manufacture materials with lower added value (e.g., plastic bags, fibers).^10,11^ However, a more attractive method in the PET recycling process is known as chemical or tertiary recycling. This method involves transforming the polymer chain through depolymerization, allowing for the recovery of monomeric units. These monomers can then be used to manufacture new virgin polymers.^12–15^ Finally, quaternary recycling or energy recycling involves the incineration of PET waste. This process is recommended for contaminated materials (e.g., hospital waste) that cannot be reused due to the high contamination risk. In this case, the polymers are converted into thermal energy, which is then converted into electrical energy, although gases are still emitted into the atmosphere.^16–18^

Of all of the recycling methods mentioned, chemical recycling aims to achieve a circular economy by using a chemical process to recycle and recover plastic materials from the petrochemical industry. This PET waste recycling process aligns with sustainability principles because it involves total or partial depolymerization into monomers and oligomers, which can be used to produce new polymers. This turns PET waste, which has low added value, into raw material for producing new products with high economic value.^19–22^ The different chemical recycling processes can be classified into pyrolysis and solvolysis. Pyrolysis is a thermal degradation process that requires the addition of heat and occurs in the absence or deficiency of oxygen. At low temperatures, the process yields small amounts of aromatic pitches and light gases, which can serve as starting materials for polyolefins. At high temperatures, oils and gases are produced, which can be purified. However, in a rare case, monomers are recovered as the final product.^23,24^ PET degradation by solvolysis occurs in the presence of solvents and can be classified into glycolysis, methanolysis, hydrolysis, ammonolysis, and aminolysis. The products resulting from each recycling process are bis­(2-hydroxyethyl) terephthalate (BHET), dimethyl terephthalate (DMT), terephthalic acid (TPA), terephthal-diamide (TDA), and bis-hydroxyethylene terephthalamide (BHETA), respectively.^25,26^

Among these methods, the glycolysis process offers several advantages in terms of reaction conditions, yield, temperature, and scalability, making it a promising method for PET depolymerization in the industry. The monomer obtained through this process, BHET, has multiple applications. In the PET depolymerization process via glycolysis, a glycol, typically ethylene glycol (EG), is used as a nucleophilic agent to break the ester bonds in the PET chain, producing BHET.^27,28^ BHET has wide applicability in the industry due to its versatility, being incorporated in the production of resins, coatings, foams, and tissue scaffolds, thus contributing to a more sustainable approach in the material manufacture.^29,30^ Additionally, BHET can be repolymerized to obtain rPET with chemical and mechanical properties compared to virgin PET.^31,32^

Thus, the glycolysis process is widely utilized due to its simplicity and low cost. However, this is a slow process. Without the use of a catalyst, the glycolysis process does not achieve high values of PET conversion and BHET yield, producing a considerable quantity of oligomers due to incomplete depolymerization. Therefore, the challenge is to develop a depolymerization process catalyzed by efficient and stable catalytic systems in which reaction conditions (time, PET/catalyst ratio, PET/EG ratio) can be optimized.^33,34^ The first catalyst used for PET depolymerization was a metal acetate, reported by Vaidya and Nadkarni in 1989, using zinc acetate as the catalyst for the PET glycolysis process.^35^ Subsequently, other metal acetates were used, such as Pd­(OAc)_2_, Zn­(OAc)_2_, Co­(OAc)_2_, and Mn­(OAc)_2_ in postconsumer PET glycolysis into BHET.^36^ Recently, our group have pioneered the use of titanate nanotubes (TNT) as catalysts in the conventional PET depolymerization method, achieving high PET conversion (100%) and a significant yield of the BHET monomer (∼80%).^37–39^

In recent years, the utilization of microwaves (MW) as a heating source has become popular due to their rapid and selective heating capabilities. One of the main advantages of this method is the more uniform heating of the reaction medium (e.g., solution) compared to the conventional heating method by thermal conduction. The interaction of radiation with molecules causes molecular rotation and, consequently, heating of the medium in addition to increasing ionic conduction. This interaction generates uniform heating and reduces heating time, making it a faster process compared to conventional heating.^40^ The search for new nanomaterials has gained prominence in recent years due to their wide application. The microwave-assisted hydrothermal method is a technology that reduces energy costs and the synthesis time of nanostructured inorganic materials. Additionally, it is considered an interesting method due to its homogeneous heating, clean reactions, high yields, and simplicity in the preparation process.^41–43^ Stigger et al. synthesized calcium molybdate via a microwave-assisted hydrothermal method, modifying the temperature and pH of the medium, and applied these materials as photoelectrodes for Graetzel solar cells.^44^ In another study by Gularte et al., cobalt sulfide (CoS) was deposited on fluorine-doped tin oxide (FTO) using an in situ microwave-assisted chemical bath. The growth process of CoS crystals occurred directly on the FTO film in a closed system under microwave radiation.^45^ The solution heating rate and efficiency depend on the properties of the solvent used. Solvents can be classified based on the dissipation factor (tan δ): high (tan δ > 0.5), medium (tan δ ∼ 0.1–0.5), and low (tan δ < 0.1). These values are related to the ability of the solvent to convert electromagnetic radiation into thermal energy (heat). The solvent EG used for PET glycolysis has a high tan δ (1.350), making it an excellent medium for microwave heating.^46^

Studies using microwave radiation to degrade polymers have been widely conducted. Selvam et al. investigated the behavior of zinc oxide (ZnO) as an active catalyst for microwave-assisted PET glycolysis, showing that this type of heating is faster compared to conventional heating, achieving complete conversion with more than 95% yield of BHET in 45 min under ideal conditions. The equipment used in this study was a Monowave 450 microwave reactor (Anton Paar GmbH) that controls the temperature, time, and maximum power set.^47^ Mohammadi et al. used a laboratory oven (Mars 6, CE, Corp.) with a magnetron source for microwave generation (2.45 GHz, maximum power: 1600 W) to evaluate PET glycolysis using antimony­(III) oxide (Sb_2_O_3_) as a catalyst at 240 °C and 400 W microwave power. Glycolysis reactions were conducted in an EasyPrep closed Teflon vessel, placed in a microwave reactor equipped with a thermometer and a magnetic stirrer. This study resulted in an oligomer yield of 96.7%, with a 100% conversion of PET into oligomers in only 5 min at 240 °C (with a 10 min ramping time), although only 3.3% BHET was produced.^48^ Recently, Yuan et al. developed a method for PET depolymerization via microwave using zinc oxide modified with manganese oxide as a cocatalyst (Mn_3_O_4_/ZnO). This study demonstrated high efficiency in depolymerizing PET with only a 0.4 wt % ratio of the catalyst to PET at 175 °C for 5 min. The operation power was set to 200 W, resulting in 100% PET conversion and 88% selectivity to BHET. The MW irradiation system (CEM Discover SP) equipped with pressure and temperature sensors was used, operating at 300 W, with the safety pressure set at 250 psi (17.24 bar).^49^

Although titanate nanotubes (TNT) have been successfully applied to PET glycolysis, both the temperature and the reaction time still require improvement. Therefore, the use of microwaves for this reaction presents an unexplored alternative in the literature using this catalyst; the use of microwaves ensures more uniform heating, providing a more homogeneous reaction medium which can influence reaction time. Additionally, in a recent study, a comparison was made between the economic and environmental performances of BHET production from the traditional DMT route with a microwave-assisted PET glycolysis technology, and significant improvements were observed. The microwave-assisted process can have lower production costs and emissions and can achieve a reduction in the selling price of BHET by at least 44%. This highlights the potential cost-efficiency and environmental benefits of adopting microwave-assisted PET glycolysis over traditional methods.^50^

This work contributes to the study of the use of microwaves as a heating source for PET depolymerization, using a TNT-like catalyst, and compared it to the conventional heating method. This work aims to evaluate the catalytic behavior of titanate nanotubes in the depolymerization of PET via glycolysis, using microwaves as a heating source. The innovation in this study lies in the microwave equipment used for the reaction, which involves a domestic microwave adapted for synthesis using a microwave-transparent container, hermetically sealed with a metal lid. This container is inserted into the cavity of a domestic electric microwave oven. Such equipment is already established in the literature for the synthesis of inorganic materials,^42–45^ but there are no reports on PET glycolysis to date. Therefore, this work aims to contribute to the literature on the use of new microwave equipment for PET glycolysis in addition to developing strategies to reduce time using TNT as a catalyst in PET glycolysis.

## Experimental Section

### Materials

The materials used in the titanate nanotubes (TNT) synthesis and in the glycolysis reaction were ethylene glycol (EG, Synth), virgin PET (Rhopet S-80-Rhodia Ster/Mossi and Ghisolfi Group), postconsumer PET (transparent with flaker averaging 0.5–1.0 cm, SR Recycling), sodium hydroxide (97%, Moderna), and titanium dioxide (98% anatase phase, JB Química). All reactants were used as received.

### Titanate Nanotubes (TNT) Synthesis

Titanate nanotubes (TNT) were prepared using a typical process reported in the literature.^51^ In a beaker, 1.5 g of titanium dioxide (18.8 mmol) was mixed with 120 mL of 10 mol L^–1^ NaOH aqueous solution under constant magnetic stirring for 30 min at room temperature. The suspension was then transferred to an autoclave reactor and hydrothermally treated at 135 °C for 72 h. The resulting white precipitate was separated by centrifugation and washed with distilled water until the filtrate reached pH = 8. Finally, the precipitate was dried at 80 °C for 24 h.

### Characterization of Titanate Nanotubes (TNT)

The structural parameters of the TNT powder were analyzed by X-ray diffraction (XRD) measurements (Shimadzu XRD 7000) using copper Kα radiation (λ = 1542 Å), 40 kV, 30 mA, within a 2θ range of 5–70°, with a scan speed of 0.02° min^–1^, and a counting time of 2.0 s. The size and shape of TNT were analyzed by field scanning electron microscopy (SEM, FEI Inspect F50) in SE mode (secondary electron beam). SEM images were obtained with a 10 kV tension, and the samples were analyzed in powder form. Transmission electron microscopy (TEM) images were obtained using a transmission electron microscope (FEI Tecnai G2 T20) with copper grids with carbon film (300 mesh). The average diameter of TNT (*n* = 25) was measured using the ImageJ program (Fiji). This analysis also evaluated the internal structure of the TNT, measuring the numbers of rolled and multilayer lamellar walls of the sample. The chemical structure of TNT was analyzed by Fourier transform infrared (FTIR) spectroscopy (utilizing a PerkinElmer Spectrum 100). The spectrum was obtained over the wavenumber interval of 4000–650 cm^–1^ with 12 scans and a resolution of 4 cm^–1^, using the universal attenuated total reflectance (UTAR) accessory. The samples were analyzed in powder form.

### Microwave-Assisted Glycolysis of PET

The microwave (MW) used in this study is an adaptation of a domestic microwave for synthesis, working with a maximum output power of 800 W and a frequency of 2450 GHz, as illustrated in Figure 1. The equipment operates as follows: the microwave generated by magnetron falls upon the reactor cell, where the reaction occurs. The magnetron is connected to a controller, with a thermocouple that measured the local temperature to control the temperature of the process. The pressure is self-generated in accordance with the temperature process and the reaction medium. The reactor cell and reaction cup (100 mL) are made of Teflon, and the closing system is made of stainless steel.

**Figure 1:**
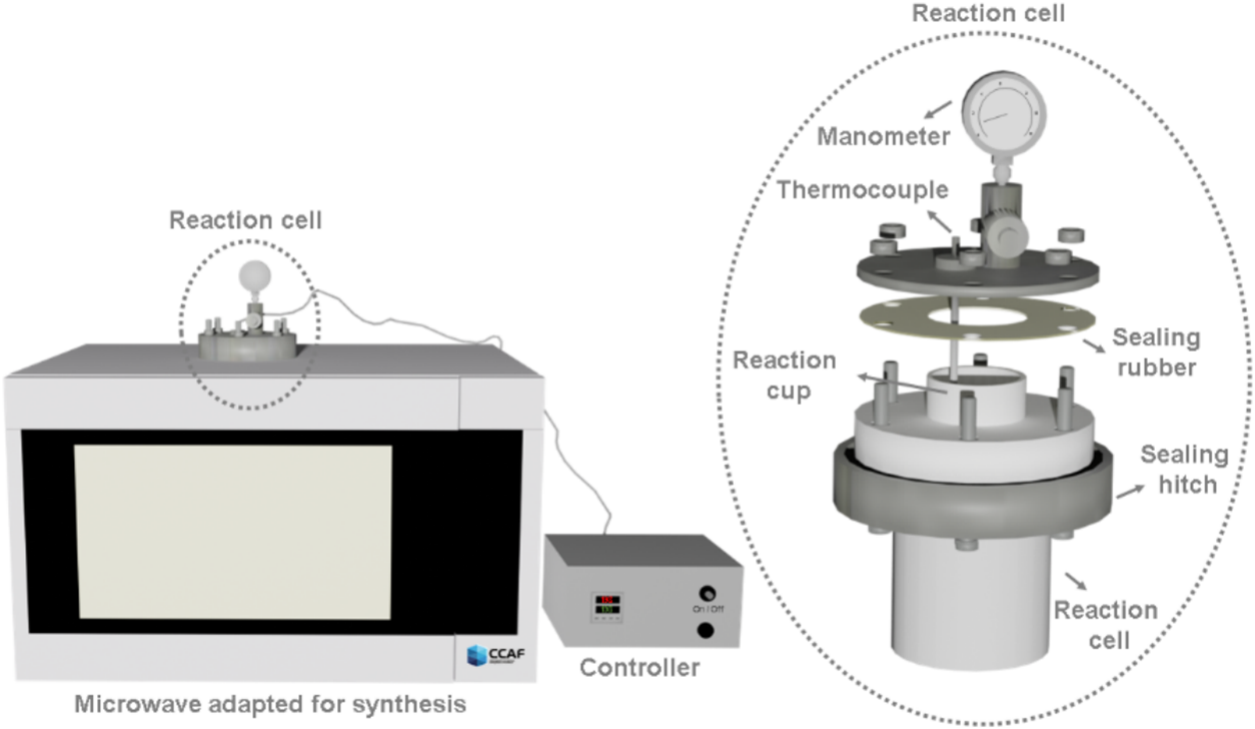
Microwave coupled reactor cell system used in this study.

For the catalytic experiments, material quantities were used, with weight ratios PET/catalyst of 300 and EG/PET of 4, according to the literature.^38^ First, the catalyst, PET, and EG were homogenized by a magnetic stirrer for 15 min. Subsequently, the suspension was transferred to the reaction cup and placed in the reaction cell, illustrated in Figure 1. The glycolysis reaction was evaluated at different times (5, 10, 15, 30, 45, 60, 120, and 180 min) at 196 °C, reaching a maximum pressure of 1 bar. After the reaction time, the reaction medium was transferred to a beaker and 100 mL of hot water was added under magnetic stirring. The suspension was then filtered under reduced pressure, and the filtrate obtained was stored at 4–10 °C for 72 h to allow BHET crystals to form. These crystals were filtered and dried at 60 °C for 24 h. This experimental process is illustrated in Figure 2. In a second step, the process was repeated using TiO_2_ as catalyst and without catalyst, with a reaction time of 60 min. This time was evaluated to achieve the total conversion of both virgin and postconsumer PET.

**Figure 2:**
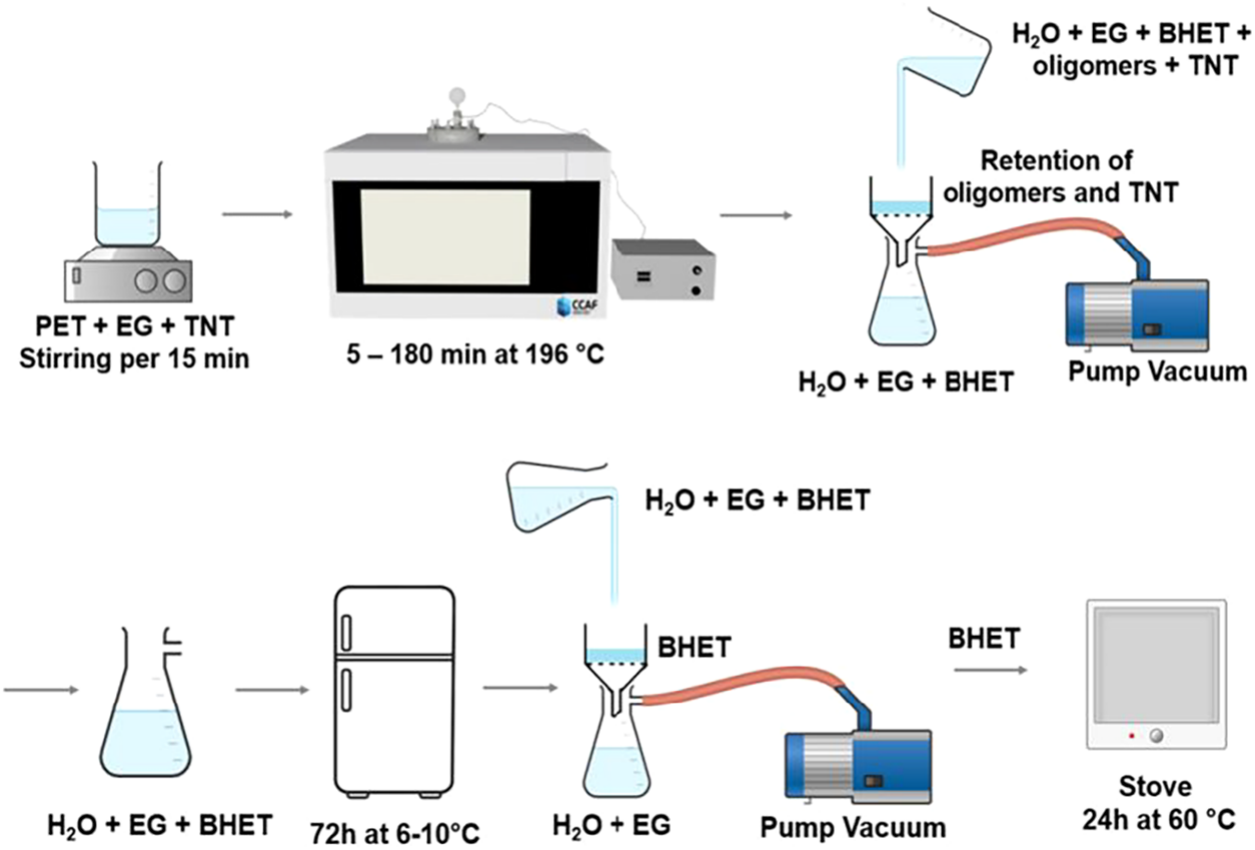
Scheme of the glycolysis process with a closed system using microwave radiation.

The BHET yield (*Y*, %) produced by depolymerization of PET was calculated according to eq 1:^30,39,52^

1 $$Y=\frac{\frac{W_{\mathrm{BHET},f}}{{\mathrm{MW}}_{\mathrm{BHET}}}}{\frac{W_{\mathrm{PET},i}}{{\mathrm{MW}}_{\mathrm{PET}}}}\times 100$$

where *W*_BHET,f_ represents the final weight of BHET, MW_BHET_ represents the molar weight of BHET (254 g mol^–1^), *W*_PET,i_ represents the initial weight of PET used in reaction, and MW_PET_ represents the molar weight of the repeat unit of PET (192 g mol^–1^). The PET conversion (*C*, %) by glycolysis reaction was calculated according to eq 2:^30,39,52^

2 $$C=\frac{W_{i}-W_{f}}{W_{i}}\times 100$$

where *W*_i_ represents the initial weight of PET and *W*_f_ represents the weight of undepolymerized PET.

### Products of PET Glycolysis

Thermal stability of products obtained from the depolymerization of virgin and postconsumer PET was analyzed by thermogravimetric analysis (TGA, in a Leading Thermal Analysis, model SDT Q600 from NETZSCH) in the range from 30 to 600 °C at a heating rate of 10 °C min^–1^ under inert atmosphere of N_2_. To complement the thermal evaluation, BHET, oligomers, and PET samples were subjected to differential scanning calorimetry (DSC, model Q2000 from TA Instruments), from ambient temperature to 350 °C at a heating rate of 10 °C min^–1^ under an inert atmosphere of N_2_. Furthermore, the chemical structure of the PET glycolysis products was evaluated using Fourier transform infrared (FTIR) spectroscopy (PerkinElmer Spectrum 100). The samples were analyzed in powder form. The spectra were obtained over a wavenumber range of 4000–650 cm^–1^ with 12 scans and a resolution of 4 cm^–1^, using the universal attenuated total reflectance (UATR) accessory. Additionally, the molecular structure and chemical composition of the BHET product were evaluated by nuclear magnetic resonance spectroscopy (NMR, Bruker Ascend 400 NMR spectrometer). The samples were solubilized in deuterated dimethyl sulfoxide (DMSO).

## Results and Discussion

### Characterization of TNT

To evaluate the formation of TNT, XRD analysis was performed. Figure 3a shows the X-ray diffraction patterns for the titanate nanotube (TNT), where the identified peaks are situated at 2θ = 10° (200), 24° (110), 28° (211), 48° (020), and 62° (422). The peak situated at 2θ = 10° represents the interlamellar distance; the peaks 2θ = 24 and 28° refer to diagonal planes; the peak 2θ = 48° corresponds to the planes formed by the TiO_6_ octahedral; and the peak at 62° indicate the intercalation of Na^+^ ions between the lamellar structure. These results are consistent with the literature^53–56^ and confirm the TNT’s crystallinity. From the Bragg’s equation, the interlayer distance was calculated to be 0.87 nm, similar to values reported in the literature.^37,57^

**Figure 3:**
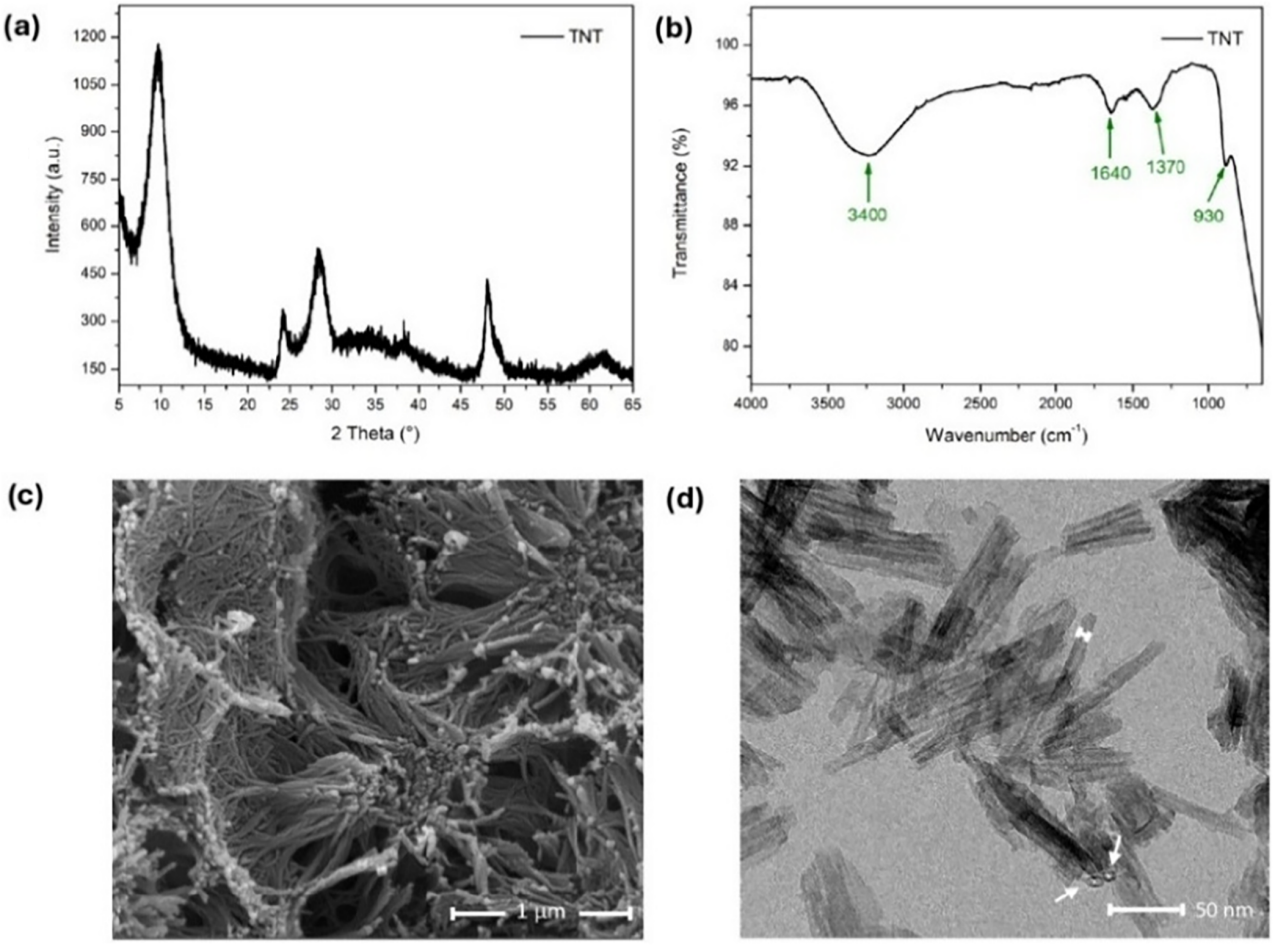
Diffractogram (a); FTIR spectrum (b); and SEM (c) and TEM (d) images of TNT obtained by the hydrothermal method.

The chemical structure of the TNT was evaluated by Fourier transform infrared (FTIR) spectroscopy. The spectrum (Figure 3b) was obtained in the 4000–650 cm^–1^ range. The characteristics bands reported are the first located at 3400 cm^–1^, indicative of hydroxyl groups (Ti–OH) adsorption on the surfaces; the second band at 1640 cm^–1^, attributed to the vibrations of the H–O–H bond deformation; and the last band at 930 cm^–1^, referring to the Ti–O vibration, which coordinated sodium ions.^58–60^ Additionally, the new peak found at 1370 cm^–1^ is attributed to the protonation of the surface TiO_2_ under acidic solution, forming Ti–OH_2_^+^ groups, which confirms the formation of titanate nanotubes.^61^

To evaluate the morphology of the TNT nanostructure, SEM and TEM analyses were performed, as shown in Figure 3c,d, respectively. Figure 3c illustrates the presence of closely packed agglomeration of TNTs, each several micrometers in length. It is also possible to observe the shape of multilayered nanotubes, which are uniform and well formed with a hollow structure, open ends, outer diameter of 10 nm, and length of 70–100 nm. The size and shape are better identified by the TEM image in Figure 3d, where the uniform distribution of nanotubes is clearly visible. These morphological characteristics have been reported in other works.^62–65^

### Glycolysis Reaction and Analysis of Its Products

The microwave-assisted glycolysis of virgin and postconsumer PET was performed under the same reaction conditions. Figure 4 shows the results of PET conversion (*C*, %) and BHET yield (*Y*, %) for both types of PET using TNT as a catalyst at different reaction times (5, 10, 15, 30, 45, 60, 120, and 180 min).

**Figure 4:**
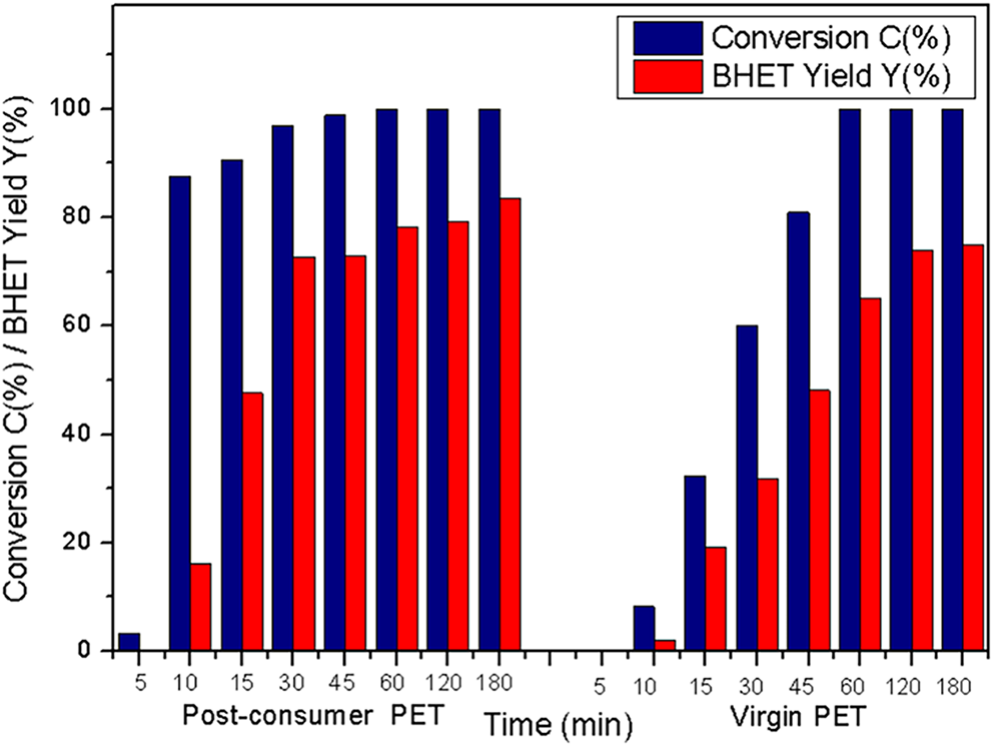
PET conversion (*C*, %) and BHET yield (*Y*, %) obtained from microwave-assisted glycolysis of virgin and postconsumer PET (reaction conditions: weight ratio EG/PET = 4:1; 1 mol % of catalyst; *T* = 196 °C).

The first interesting and expected result was the decrease in the reaction time for PET glycolysis using microwave radiation compared with the conventional method. Conventional glycolysis requires approximately 2–4 h to achieve total conversion of PET and obtain high yields in BHET.^33,38,66–68^ In our case, we achieved total conversion of PET within 60 min using microwave radiation as the heating source. This decrease in time is directly related to several factors. The first factor is associated with the self-generated pressure. It is fundamental for the system pressure to reach 1 bar to ensure complete PET conversion. It is worth mentioning, however, that the pressure does not remain at 1 bar at all times; instead, it varies between 0.5 and 1 bar according to the temperature adjustment made by the microwave system. Furthermore, it was noted that in some reactions where the pressure did not reach the maximum value, total depolymerization of PET did not occur. This confirms the importance of the system pressure for total depolymerization. Studies show that the pressure improves the PET depolymerization reaction, allowing for greater BHET yields with shorter reaction times. Additionally, as it is a closed reactor, it prevents the loss of volatile organic materials.^69–71^

The second most important factor is temperature. In our system, temperature control is managed by a controller that reads the thermocouple’s temperature and activates the electromagnetic microwave radiation. When the temperature is 3 °C below the set point, the controller triggers the magnetron, which continues emitting radiation until the temperature rises 3 °C above the set point. This behavior occurs throughout the process, and it is essential that the temperature remains around 196 °C, with a variation of approximately 3 °C. Mendiburu-Valor et al. showed in their studies that temperature and time are fundamental parameters for the depolymerization of PET. With 10 min of reaction at 220 °C under pressure, the final product was mainly BHET, while at 180 °C, it took 30 and 60 min to obtain the same BHET, indicating that depolymerization occurs at lower temperatures but requires a longer reaction time.^72^ López-Fonseca et al. evaluated the influence of temperature in the range of 165–196 °C on an EG/PET molar ratio of 7.6:1 and PET/Zn­(Ac)_2_ molar ratio of 100:1 during a 1 h reaction time for conventional PET glycolysis. They observed that conversion at 196 °C was 1.5% higher than at 180 °C, noting that 196 °C is the normal boiling point of EG.^52^

The last factor is the shape of PET, with virgin PET being in pellet and postconsumer PET in flakes. This system does not involve stirring the reaction medium, which could explain the difference in BHET yield between virgin and postconsumer PET. The results of reaction (5, 10, 15, 30, 45, 60, 120, and 180 min at 196 °C) are shown in Table 1. It can be observed that between 5 and 45 min, the conversion of postconsumer PET is higher than that of virgin PET, indicating that virgin PET takes longer to convert. Viana et al. showed in their study the relationship between the conversion and the geometry of postconsumer PET in the glycolysis reaction (180 °C and 90 min). This investigation included seven different particle sizes and demonstrated that reaction velocity is largely dependent on the contact area.^73^ Similarly, Deng et al. showed the influence of PET particle size on glycolysis and found that smaller particle size could significantly speed up reaction rate but had little effect on the yield or conversion under glycolysis condition (190 °C, EG/PET = 5:1, catalyst/PET = 1:20). They achieved 100% conversion of waste PET and a product yield 82.3% of BHET.^74^

**Table 1:** PET Conversion (*C*, %) and BHET Yield (*Y*, %) from Virgin and Postconsumer PET Glycolysis Using TNT Catalyst^a^

PET	time (min)	conversion (*C*, %)	yield (*Y*, %)
virgin	5	0.02 ± 0.01	0.06 ± 0.02
	10	8.3 ± 0.3	2.1 ± 0.2
	15	32.4 ± 0.7	19.2 ± 0.3
	30	60.1 ± 1.3	31.8 ± 0.5
	45	80.9 ± 2.2	48.1 ± 0.8
	60	100.0^d^	65.2 ± 0.1
	60^b^	31.1 ± 0.6	1.6 ± 0.1
	60^c^	27.4 ± 1.1	0.44 ± 0.04
	120	100.0^d^	73.9 ± 0.3
	180	100.0^d^	74.9 ± 0.6
postconsumer	5	3.4 ± 0.2	0.22 ± 0.05
	10	87.4 ± 1.1	16.2 ± 0.4
	15	90.6 ± 0.4	47.6 ± 0.5
	30	96.9 ± 0.5	72.7 ± 0.5
	45	98.8 ± 0.9	73.0 ± 0.7
	60	100.0^d^	78.2 ± 0.2
	60^b^	45.4 ± 0.5	1.13 ± 0.07
	60^c^	68.5 ± 0.4	0.68 ± 0.07
	120	100.0^d^	79.1 ± 0.7
	180	100.0^d^	83.6 ± 1.3

a Reaction conditions: weight ratio EG/PET = 4:1; 1 mol % of catalyst; *T* = 196 °C.

b Without catalyst.

c TiO_2_ catalyst.

d For 100% conversion values, the standard deviation is zero.

To prove the necessity and efficiency of using a TNT catalyst, PET glycolysis was evaluated over 1 h without catalyst and with TiO_2_ as a catalyst (precursor used to obtain TNT). The results of this study are shown in Table 1. The 1 h time frame was chosen because it was the time needed for both virgin and postconsumer PET to reach 100% of conversion. In the glycolysis reaction of both PET types carried out without a catalyst, BHET yields were below 2% and PET conversions below 45%. Similarly, when TiO_2_ was used as a catalyst, BHET yields were below 1% and PET conversions were below 69%. In both cases, partial depolymerization of PET occurred, resulting in the formation of oligomeric and polymer chains instead of monomers, leading to low BHET yields.^7,28,75^ Llopis et al. evaluated the partial depolymerization of PET glycolysis using an excess of ethylene glycol in the absence of catalyst at different times (from 10 to 120 min) and temperatures (from 160 to 210 °C). They observed that temperature played a critical role in promoting surface PET degradation processes.^76^

Table 2 lists some literature results obtained from PET depolymerization via glycolysis. It is observed that regardless of the catalyst, the glycolysis process using microwave radiation as a heating source significantly reduces the reaction time compared to the conventional heating method. Another point to highlight is the use of the system under pressure, which also reduces the reaction time compared to conventional heating.

**Table 2:** Comparison of Postconsumer PET Glycolysis with Different Catalysts

catalyst	temperature (°C)	EG/PET (w/w)	PET/catalyst (w/w)	time (min)	PET conversion (*C*, %)	BHET yield (*Y*, %)	ref
zinc acetate	196	5:1	100:1	35	97.1	78.0	^77^
ZnO	210	10:1	100:1	45	100.0	95.0	^47^
titanium phosphate (TiP)	200	2.77:1	500:1	45		87.4	^33^
CoFe_2_O_4_	200^a^	5:1	100:1	60	100.0	95.4	^78^
10%Ce/Al_2_O_3_	250^a^	6:1	37.5:1	60	100.0	70.0	^13^
FAU-type zeolite (100-FAU)	196^b^	6.3:1	50:1	90	100.0	72.1	^79^
ultrasmall cobalt nanoparticles	180^b^	27.5:1	66.7:1	180	96.0	77.0	^80^
TNT	196^b^	4:1	300:1	120	99.0	76.7	^38^
TNT	196^b^	4:1	300:1	180	99.0	73.2	^38^
TNT	196	4:1	300:1	30	96.9	72.7	this work
TNT	196	4:1	300:1	45	98.8	73.0	this work
TNT	196	4:1	300:1	60	100.0	78.2	this work
TNT	196	4:1	300:1	120	100.0	79.1	this work
TNT	196	4:1	300:1	180	100.0	83.6	this work

a Heating: microwave; under pressure.

b Heating: microwave; conventional.

Lima et al. used TNT as a catalyst in the depolymerization of PET via glycolysis using conventional heating, with a reaction time of 180 min, achieving a BHET yield of 73.7% for a 100% PET conversion.^38^ In comparison, our work achieved a BHET yield of 78.2% for a reaction time of 60 min, and our work demonstrated a reduction of one-third of the time required to 100% of PET conversion. These results demonstrate that the microwave system presented in this study is efficient for PET glycolysis, yielding results comparable to those in the literature using the same catalyst while significantly reducing reaction times compared to the conventional heating method.

When comparing our work to the other studies, we can highlight the advantages of microwaves in the glycolysis of PET. Other studies have evaluated niobium-based catalysts, achieving 100% PET conversion and 85% BHET yield at 195 °C in 220 min using the conventional method.^28^ Additionally, catalysts of the Mg-AlO@Fe_3_O_4_ type have achieved a BHET yield of more than 80 mol % at 240 °C for 90 min.^19^ In contrast, our work demonstrates that microwaves in PET glycolysis are a faster method compared to the conventional method. This highlights the efficiency and potential of microwave-assisted glycolysis for PET recycling.

### Kinetic Study

The depolymerization of PET is a complex reaction in which chemical interactions and mass transfer phenomena occur simultaneously, depending on the type of catalytic system used (homogeneous or heterogeneous).^81–83^ The homogeneous kinetics of PET depolymerization can be described by the power-law model, where there is almost no phase change occurring in the molten state. However, this model is not applicable in the glycolysis process at low temperatures due to the existence of phase interfaces.^84^ Schlüter et al. used the biomass-derived green platform molecule *y*-valerolactone (GVL) as an additional cosolvent in the traditional PET reaction mixture to improve kinetics. The reaction kinetics were evaluated by kinetic constants obtained from the pseudo-first-order reversible reaction model. In their study, they used a reference kinetic data set to determine PET glycolysis catalyzed by ZnAc_2_ at 190 °C and 1 bar.^83^

As in previous studies,^85,86^ our study was carried out at a temperature far below the melting point of PET. Initially, the system is a suspension of PET and catalyst (heterogeneous system). The reaction occurs on the surface of the PET particles, and this surface changes as the conversion to the product increases, leading to the end of reaction when these particles disappear.^87^ On the other hand, as the reaction occurs on the surface of the PET particles, the process of breaking the PET chains progresses from the surface into the internal bulk of the particle, producing oligomers or monomers.

Kinetic analysis is extremely important to understanding the general behavior of the PET glycolysis reaction. For the depolymerization of PET, a simple kinetic model should be used, represented by a pseudo-first-order reaction. In this model, the depolymerization reaction rate is proportional to the concentration of PET and EG,^84,88,89^ according to eq 3:

3 $$\frac{d\left[\mathrm{PET}\right]}{dt}=-k\left[\mathrm{EG}\right]\left[\mathrm{PET}\right]$$

where [PET] is the PET concentration; [EG] is the EG concentration and *k* is the kinetic constant. Under the experimental conditions proposed in this work, the EG is in excess, thus rewriting eq 3:

4 $$\frac{d\left[\mathrm{PET}\right]}{dt}=-k^{\prime }\left[\mathrm{PET}\right]$$

considering [PET] = [PET]_0_(1 – *x*), we can rewrite eq 4:

5 $$\frac{d\left[X\right]}{dt}=-k^{\prime \prime }\left(1-x\right)$$

where *x* is the PET conversion, [PET]_0_ can be rewritten as *k*″, which is a constant in the pseudo-first-order reaction. Finally, integrating eq 5 gives

6 $$\ln\left(\frac{1}{1-x}\right)=-k^{\prime \prime }t$$

Figure 5a shows the relationship between PET conversion (virgin and postconsumer) and reaction time. To determine the kinetic behavior of the PET reaction in this system, eq 6 was applied using the conversion values corresponding to 10, 15, 30, and 45 min. This data was used to plot ln­(1/(1 – *x*)) vs time of PET glycolysis, as shown in Figure 5b. The conversion values at 5 min were low and therefore disregarded. Furthermore, after 60 min of reaction, the conversion reached 100%, so this time was not used for kinetic calculation.

**Figure 5:**
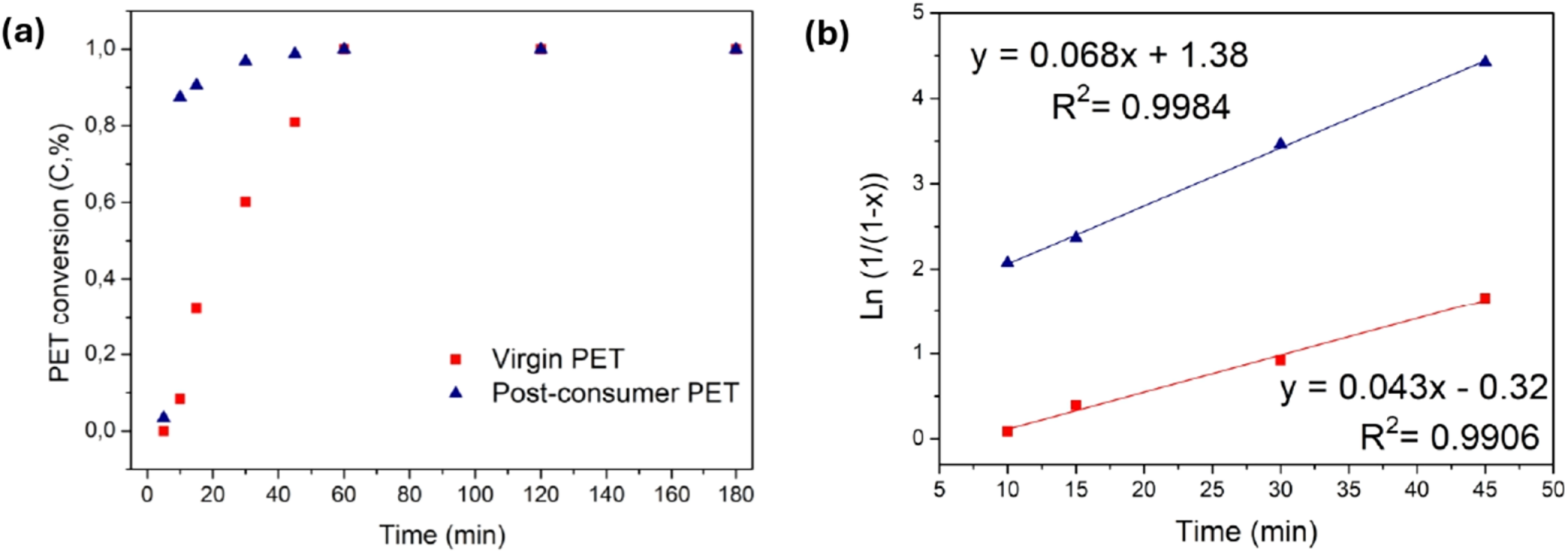
(a) Virgin and postconsumer PET conversion and (b) effect of type on the rate of PET glycolysis.

The *R* ^2^ values indicate linear correlation coefficients of the kinetic reaction, both higher than 0.99. This confirms that the glycolysis process using the pseudo-first-order equation is satisfied.^86^ The rate constants were obtained from the slope of the straight lines, with values of 0.043 and 0.068 min^–1^ corresponding to the depolymerization of virgin and postconsumer PET, respectively. To date, there are no kinetic studies of PET depolymerization using TNT as a catalyst. For comparison, Javed et al. evaluated the kinetics of glycolytic depolymerization of postconsumer polyethylene in the presence of sodium ethoxide (EtONa), obtaining a slope of 0.0307 and an *R* ^2^ of 0.9822 at 197 °C, evaluated in the conventional glycolysis process.^90^ In another study, Pham et al. evaluated the behavior of MOF-based catalysts in traditional PET glycolysis and found that all the curves exhibited linear coefficients *R* ^2^ higher than 0.98.^91^

### Analysis of Glycolysis Products

The products obtained from the glycolysis of virgin and postconsumer PET at 30, 60, and 180 min were evaluated by FTIR, DSC, TGA, ^1^H NMR, and ^13^C NMR. Figure 6 shows the FTIR spectra of virgin and postconsumer PET, oligomers, and BHET monomer. The characteristic absorption bands of PET can be identified in 1712 cm^–1^, attributed to the stretching of the carbonyl group, characteristics of the ester group, indicating the connection of ketones (C=O).^92,93^

**Figure 6:**
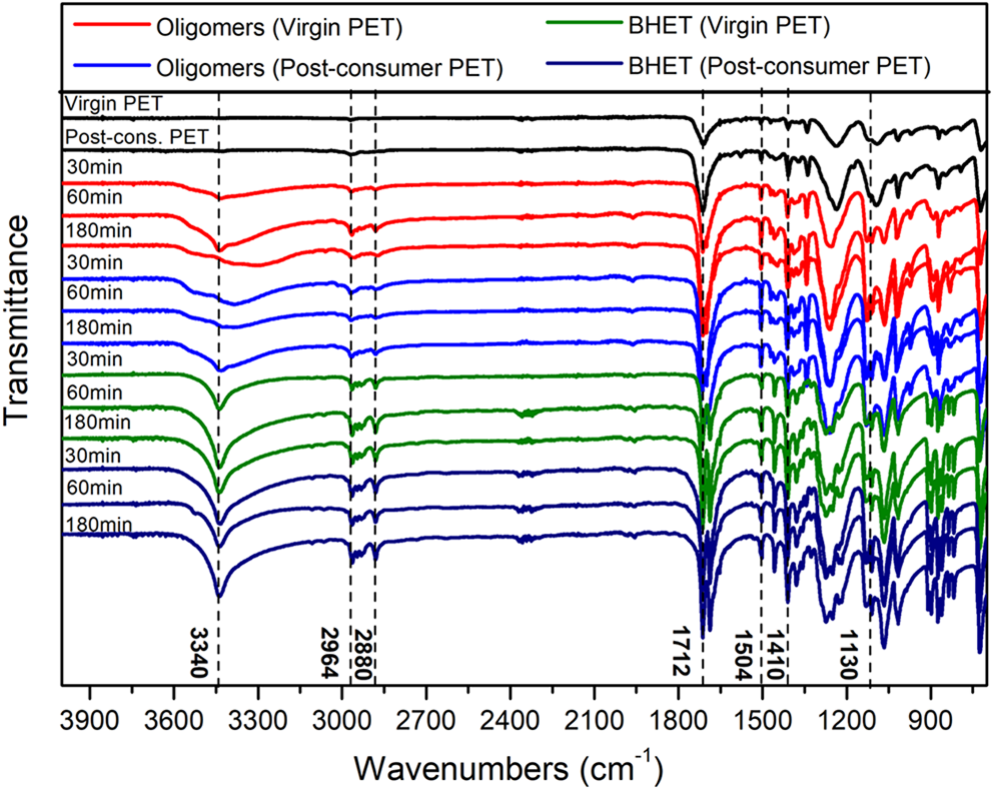
FTIR spectra of PET, oligomers, and BHET.

The band at 3340 cm^–1^ attributed to the stretching of the hydroxyl group is an important signal in the spectra that differentiates the BHET monomer from the oligomers and the starting PET. In the spectra of BHET (from the virgin and postconsumer PET), a narrow band is observed, while a broad, low-intensity band appears in the oligomers spectra, indicating the mixture of oligomers formed. However, this band is absent in PET spectra.^48,94,95^ The characteristic bands located at 2967 and 2879 cm^–1^ are assigned to the symmetric and asymmetric stretching of the C–H bond, respectively. The bands at 1412 and 1504 cm^–1^ are attributed to the C–H bonds of the aromatic ring and are more noticeable in the BHET monomer spectra, as it is a smaller molecule.^7,96–98^

The BHET formation at different depolymerization times was evaluated by ^1^H NMR and ^13^C NMR (Figure 7). Figure 7a shows the characteristic peaks of BHET obtained from virgin and postconsumer PET depolymerization. Peak 1 is attributed to aromatic ring protons (δH = 8.13 ppm, s, 4H), peak 2 to protons of the hydroxyl group (δH = 4.97 ppm, t, 2H), peak 3 to methylene protons adjacent to the −OH group (δH = 3.73 ppm, m, 4H), and peak 4 to protons of methylene adjacent to the −COO– group (δH = 4.33 ppm, t, 4H). These results align with the literature.^99,100^ The peak located at 2.51 ppm refers to the deuterated solvent used (DMSO), and the peak located at 3.34 ppm can be attributed to residual H_2_O.^97,101^ Figure 7b shows the signals corresponding to the carbons of the BHET chemical structure: peak 1 is attributed to carbon of the ester groups −COO– (δC = 165.6 ppm), peak 2 to carbon of the aromatic carbon linked to the ester carbonyl groups (ph-COO) (δC = 134.2 ppm), peak 3 to aromatic carbon of the benzene ring (δC = 129.9 ppm), and peak 4 (δC = 67.4 ppm) and peak 5 (δC = 59.4 ppm) to methylene carbon (COO–CH_2_ and −CH_2_–OH). The deuterated solvent’s peak appears at 40 ppm.^102,103^

**Figure 7:**
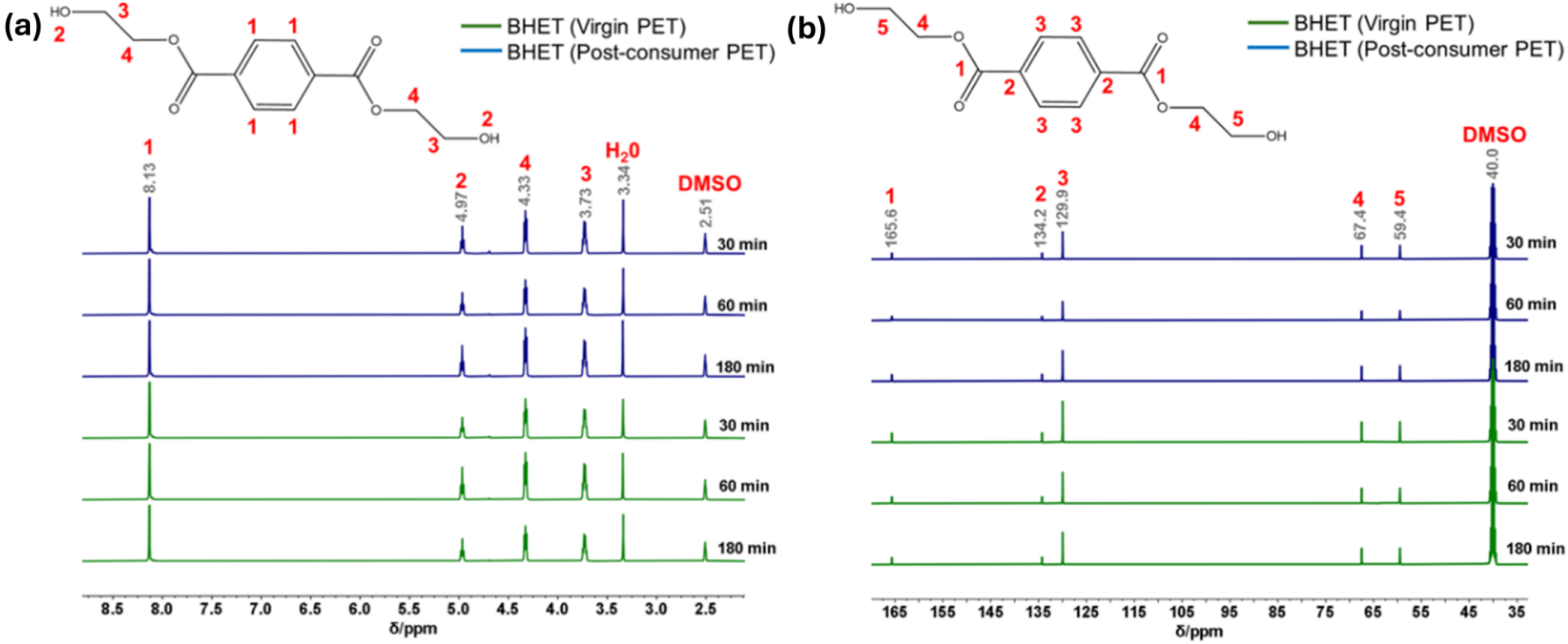
^1^H (a) and ^13^C (b) NMR spectra of BHET obtained by PET glycolysis.

Figure 8a shows the TG curves of PET, oligomers, and BHET samples obtained from PET glycolysis. For the BHET samples, two significant weight loss events are observed. The first occurs between 185 and 210 °C, with a weight loss of around 25–30%, corresponding to the thermal degradation of the BHET. The second event occurs around 385–430 °C, with a weight loss of around 65–70%, attributed to degraded PET.^104–106^ This PET can be explained by the thermal polymerization process of BHET forming PET during the TGA analysis.^10,102^ The oligomers, as byproducts of glycolysis, show similar behavior to BHET with two weight loss events. However, in this case, the first weight loss is less significant, around 10–15%, indicating the presence of BHET in the byproducts.^107^ This event is better observed in Figure 8b, which presents a zoomed-in view of this region of the curve. The second event, around 385–430 °C, with a weight loss of around 85–90%, is also attributed to PET degradation formed during the analysis. Another point to highlight is that regardless of the PET depolymerization time (30, 60, or 180 min), all samples showed the same behavior.

**Figure 8:**
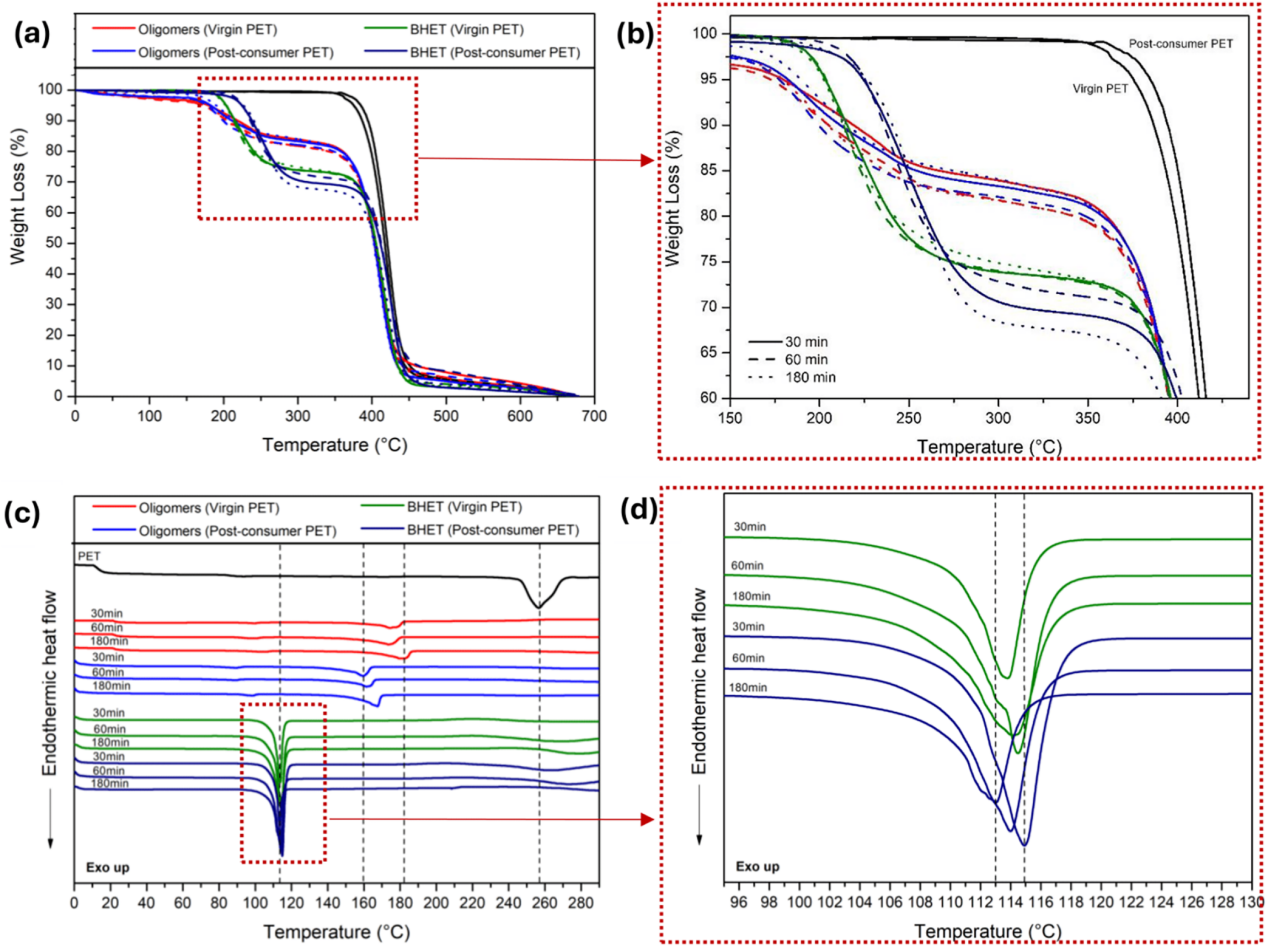
TG (a, b) and DSC (c, d) curves of BHET, oligomers, and PET.

To evaluate the melting temperatures of BHET and oligomer products, DSC analysis was performed. Figure 8c shows DSC curves of PET, oligomers, and BHET products obtained by microwave-assisted PET depolymerization. An endothermic peak between 110 and 120 °C was observed for all BHET products evaluated, which was assigned to the melting point of this monomer.^13,102,108^ Figure 8d shows a small increase in the BHET melting temperature as the glycolysis time of virgin PET increases; however, the opposite behavior occurs for postconsumer PET. Furthermore, less intense melting peaks at approximately 170 °C were observed in oligomer products but were absent in the BHET analysis, indicating a higher purity of the produced BHET.^69,73^ Finally, a small endothermic event occurs around 228–265 °C in the BHET samples, attributed to impurities and vaporization of materials present in the PET. The peak at around 256 °C is attributed to the melting of solid PET, which can be explained by the thermal polymerization process of BHET forming PET again.^103,109^

Based on DSC and TGA analyses of the products generated from virgin and postconsumer microwave-assisted PET glycolysis, it was concluded that the purified monomer was BHET, irrespective of the PET glycolyzed.

## Conclusions

This work successfully demonstrates the efficiency of a domestic microwave adapted for PET depolymerization via glycolysis using a closed system under pressure. This new microwave model for chemical recycling of PET achieved a significant reduction in reaction time compared to the traditional method using titanate nanotube as a catalyst. This MW system, not yet reported in the literature for PET glycolysis, presented interesting results and contributed to advances in research on heterogeneous catalysis. It achieved 100% PET conversion within 60 min and a BHET yield close to 80%, using an EG/PET = 4:1; PET/catalyst = 300:1; *T* = 196 °C.
